# Burden of infectious diseases among undocumented migrants in France: Results of the Premiers Pas survey

**DOI:** 10.3389/fpubh.2022.934050

**Published:** 2022-08-04

**Authors:** Nicolas Vignier, Sohela Moussaoui, Antoine Marsaudon, Jérome Wittwer, Florence Jusot, Paul Dourgnon

**Affiliations:** ^1^Institut de recherche et documentation en économie de la santé, IRDES, Paris, France; ^2^Centre d'Investigation Clinique Antilles Guyane, CIC INSERM 1424, Centre hospitalier de Cayenne, Cayenne, French Guiana; ^3^Sorbonne Université, INSERM UMR 1136, Institut Pierre Louis d'Épidémiologie et de Santé Publique, IPLESP, Department of Social Epidemiology, Paris, France; ^4^French Collaborative Institute on Migration, Institut Convergences Migrations, ICM, Aubervilliers, France; ^5^Université Sorbonne Paris Nord, UFR SMBH, IAME, INSERM UMR 1137, Hôpital Avicenne, Hôpitaux Universitaires Paris Seine-Saint-Denis, AP-HP, Department of Infectious and Tropical diseases, Bobigny, France; ^6^Bordeaux University, Bordeaux Population Health, INSERM U1219, Economic and Management of Healthcare Organizations Team, Emos Team, Bordeaux, France; ^7^Université Paris-Dauphine, PSL-Research University, Leda-Legos, Paris, France

**Keywords:** migrants, undocumented migrants, infectious diseases, HIV, dental infection, France, HBV

## Abstract

**Introduction:**

An increase in migration rates to the European Union has been observed over the last few years. Part of these migrants is undocumented. This work aimed to describe the reported frequency of infectious diseases and their associated factors among unselected samples of undocumented migrants in France.

**Methodology:**

The Premier Pas survey is a cross-sectional epidemiological survey of a random sample (two-stage sample design) conducted among undocumented migrants recruited in Paris and the Bordeaux region, in places and facilities likely to be frequented by undocumented migrants. The percentages were weighted. The analysis was performed using Stata 15.1 software.

**Results:**

A total of 1,223 undocumented migrants were recruited from 63 places and facilities, with a participation rate of 50%. Most of them were between 30 and 40 years of age (36%), 69% were men, aged mainly 30–40 (36%) years old, from sub-Saharan Africa (60%) or North Africa (25%), and 60% had arrived <3 years earlier. Among the participants, 24.8% declared a poor perceived health status and 33.5% a chronic health condition. Dental infections concerned 43.2% of the participants. Apart from dental issues, 12.9% reported suffering from at least one infectious disease: HIV infection (3.5%), chronic hepatitis B virus infection (3.1%), upper respiratory tract infection (1.7%), skin mycosis (1.2%), skin and soft tissue infection (0.8%), chronic hepatitis C infection (0.8%), urinary tract infection (0.7%), lower respiratory tract infection (0.7%), scabies (0.3%), tuberculosis disease (0.2%), vaginal mycosis (0.6%), and herpes (0.1%). Regarding HIV, HBV, and HCV infections, 56, 71, and 89%, respectively, were diagnosed after their arrival. Chronic viral infections were more often reported by undocumented migrants from sub-Saharan Africa and Latin America. In multivariate analysis, a higher risk of reporting chronic viral infection was observed among people food insecure.

**Conclusion:**

This original study on a large random sample confirms the frequency of infectious diseases among undocumented migrants in France and the importance of integrating their screening during a health Rendezvous and their management into early access to care and inclusive medico-psycho-social management.

## Introduction

The international organization of migration (IOM) defined a migrant as a person who moves away from his or her place of usual residence, whether within a country or across an international border, temporarily or permanently, and for a variety of reasons (1). With 281 million migrants in the world in 2020, ~3.6% of the world population living outside their usual residences (2). Increased rates of migration to the European Union were observed over the last few years. Currently, ~10% of the population of the WHO European Region is estimated to be migrants (3). Migration is a growing phenomenon, influencing the health and development of both migrant and host communities.

Migrants generally do not represent a threat to the health of the host population (4). However, some subgroups of migrants are particularly vulnerable to infectious diseases and may be less well-cared for as compared to the host population (4–8).

Part of these migrants is undocumented [i.e., persons born abroad, of foreign nationality, and who have no right of residence in the country of residence (9, 10)]. Limited data are available in Europe and France on the health of undocumented immigrants (9, 11, 12). It was estimated that 1.9–3.8 million undocumented migrants live in the European Union (EU) (11). Despite the existence in France of social assistance for health cover for undocumented migrants (the State Medical Aid, AME), it is estimated that 49% of eligible undocumented migrants have not applied for their rights in Paris and the Bordeaux area (13). There were 315,000 AME beneficiaries in 2019. Several studies in Europe highlight their poor perceived health, poorest health-related quality of life, poor mental health, food insecurity, and cumulative health problems, namely, metabolic, musculoskeletal, and digestive disorders (14–16).

According to the data from the healthcare system and NGOs, undocumented migrants are unequally affected by infectious and tropical diseases in relation to the epidemiology in the countries of origin and poor living conditions in the host countries (11, 17, 18). A recent study observed an increase in ICU admissions in France for undocumented migrants, who were younger and more severely ill than other patients admitted (19).

However, no study has assessed the frequency of these infectious diseases in an unselected sample of undocumented migrants. Studies, to date, rely only on data collected in healthcare places dedicated to vulnerable populations. We improve upon the existing dataset by collecting information on various places of assistance (e.g., administrative, health, cultural, food, and housing) dedicated to vulnerable populations (13, 20, 21). This study, based on multidisciplinary approaches, aims to better understand the experience of undocumented migrants living in France regarding their health status and access to rights and healthcare.

The present work aimed to describe the reported frequency of infectious diseases, the location of their diagnosis, and their associated factors among undocumented migrants in France.

## Methods

### Type of study and sampling

The Premier Pas survey is a cross-sectional epidemiological survey of a random sample (two-stage sample design) of undocumented migrants recruited in Paris and the Bordeaux area in places and facilities likely to be frequented by undocumented migrants, between February and April 2019 (20). A first sampling constituted a sample of places and structures likely to be frequented by undocumented immigrants. A sample of organizations was subsequently drawn from the list compiled in the first stage. Information on attendance was collected in a pre-survey of the facilities. The “Structures” survey was conducted by telephone with 736 sites and organizations. The list comprised sites and organizations in central Paris, Bordeaux, and communes in the city's suburbs (Cenon, Lormont, Mérignac, Pessac, and Talence). It was compiled from sources available to the migrants, and listed in local resources for deprived individuals and migrants. These public sources were complemented by several associative sources that provided a broad representation of the services available to the individuals studied in the survey. This approach was not intended to be comprehensive; it was reproducible and was based on identical sources in Paris and Bordeaux; 87% of the organizations contacted, that catered to undocumented immigrants, agreed to participate in the survey. In the sample of surveyed organizations, we selected all the 113 organizations that declared they had at least 20 eligible users a week in the “Structures.” A total of 63 (56%) of the 113 selected organizations agreed to have the survey conducted on their premises.

In a second stage, organizations were sampled, asked to participate, and if they agreed, a sample of undocumented immigrants was recruited in the selected places. Inclusion criteria were to be undocumented, living in France and to be over 18 years old. Fourteen different languages were used to translate the questionnaire and interact with participants. The detailed methodology has been described elsewhere (15, 16). The structures included “Espace Solidarité Insertion” which is a structure offering a daytime shelter and social and health services to people living on the streets of Paris, NGOs, free outpatient health clinics (“Permanence d'Accès aux Soins de Santé”), which are mostly inside hospitals and provide free medical care and social assistance to vulnerable populations; the local health insurance centers (“Caisses Primaires d'Assurance Maladie”), which operates at a departmental level and deal with several benefits/allowances for insured people such as sickness, pregnancy, invalidity or death, and shower baths; the free health centers of Doctors of the Word NGO (“CASO”); the Point of Access to Rights (“Point d'accès aux droits”), which is a free and permanent reception area providing information, orientation, and local advice in response to legal or administrative problems; and maternal and child protection centers (“Protection Maternelle et Infantile”), which is a departmental service responsible for ensuring the health protection of mothers and children and other places. The Paris region was chosen because it is the region that faces most of the new migrant arrivals in France and where the accommodation and support structures are congested. Bordeaux is a place of arrival for migrants and adds to the study the representativeness of a small metropolis concerned by migration, with probably more means to accompany them.

### Data collection

In total, 21 interviewers were recruited and trained directly by the team that designed the survey, 18 of whom covered the Paris area and 3 of whom covered the Bordeaux area. All of them spoke English and more than 75% of them also spoke another language in which they were able to conduct the questionnaire. Most of the interviewers were social sciences graduates. A complete range of socioeconomic indicators was collected, namely, age, gender, social characteristics, migration characteristics, social isolation, material living conditions, access to care, and the place of recruitment. Educational level was defined as the number of years at school. In addition, participants were asked if they were currently experiencing infectious diseases or other health conditions. Questions about health problems were understood at the time of the survey. If the individual answered yes to one of the conditions, more specific questions were asked to specify the disease or the symptom. For each condition, the time of diagnostic before or after the arrival in France and diagnostic modalities were assessed. Dental pain and missing or broken teeth or infected teeth (cavities) status were collected. Participants were also asked if they had skin or gynecological problems. A detailed list of infectious diseases was also sought.

### Data analysis

First, we described the prevalence of infectious diseases by frequency order using percentages. Infectious diseases have been validated and reclassified by senior medical doctors and specialists in infectious and tropical diseases. The major diseases were compared by gender using a chi-square test. Data were weighted based on the probability of inclusion. The weighting considered the sampling plan and types of organizations and services provided to users. The collection of each respondent who visits the participating organizations enabled multiple inclusions to be considered in the weights' calculation. We calculated survey weights by taking into account the facility type, attendance, and type of services provided. More precisely, we collect the weekly average number of undocumented immigrants attending each of these places. For each place, we then weight undocumented immigrants according to this average number. That is, we compensate for a deviation from this average by giving higher (lower, respectively) weights for those that are below (above, respectively) the weekly average. The percentages were weighted using those weights. The percentages presented are based on the data reported. Numbers without missing data are systematically reported.

Then we performed univariate and multivariate analyses using logistic regression for having declared at least one chronic viral infection (HIV, HBV, or HCV). Associations were expressed using odds ratios (ORs) with 95% CIs. The multivariate analysis by logistic regression used a stepwise backward variable selection method with a *p*-value threshold of 5%. All analyses have been performed with Stata© 15.1 software (StataCorp, College Station, TX, USA).

### Ethics and funding

Participation in the survey was free and anonymous. No information was transmitted to any administration. The information collected in the survey was only used by researchers. The study was carried out by the French Institute for Research and Documentation in Health Economics (IRDES, Institut de recherche et documentation en économie de la santé) under the supervision of the French Data Protection Authority (CNIL), which guarantees respect for anonymity, privacy, and the protection of personal data. The project was supported by the French National Research Agency (Agence Nationale de la Recherche, ANR) through the 2015 Generic ANR Call for Projects.

## Result

A total of 1,223 undocumented migrants were recruited in Paris (86%) and the Bordeaux (14%) area, in 63 places and structures (selected from 736 places and structures listed). The places of recruitment were associations (44%), solidarity and integration spaces (12.7%), shower-baths (9.5%), free health centers (9.5%), health centers (9.5%), and health insurance centers (6.3%), Doctor of the World NGO (3.2%), maternal and child protection centers (3.2%), social action centers (1.6%), family planning centers (1.6%), and access to rights points (1.6%). The participation rate of the organizations approached was 56%, and of eligible migrants was 49%. The interview was mainly conducted in French (75%), English (7%), Arabic (8%), or Spanish (4%). The language barrier was an obstacle in only 5% of the cases.

The sample was composed of 69% men, aged mainly 18–30 (32%) or 30–40 (36%) years old participants, from sub-Saharan Africa (60%), North Africa (25%), or Latin America (7%); 27% had arrived in France less than a year before, 33% had arrived in France 1–3 years before, and 26% had arrived in France more than 5 years before (Table 1).

**Table 1:** Sociodemographic characteristics of undocumented migrants in Paris and Bordeaux, Premier Pas survey (*n* = 1.223).

	**%**
**Age**	
18–30 years	32
30–40 years	36
40–50 years	21
50–60 years	8
60 years old and over	3
**Gender**	
Female	31
Male	69
**Length of stay in France**	
<3 months	15
3 months to 1 year	23
1–3 years	28
3–5 years	12
More than 5 years	22
**Reasons for coming to France**	
Health-related reasons	10
Economic reasons	47
Political reasons	22
Family-related reasons	8
Private reasons	14
Educational reasons	5
**Region of birth**	
Sub-Saharan Africa	60
North Africa	25
Latin America and US	7
Asia	5
Europe	3
**Work activity**	
Active	25
**Educational level**	
No school education	12
Under 12 years	9
12–19 years	39
19 years and over	39
Student	1
**Type of housing**	
Personal housing	7
Living with a relative	25
Shared accommodation	11
Reception center	11
Hostel	9
In a hotel	12
Temporary housing, living on the street	26

**Figure 1:**
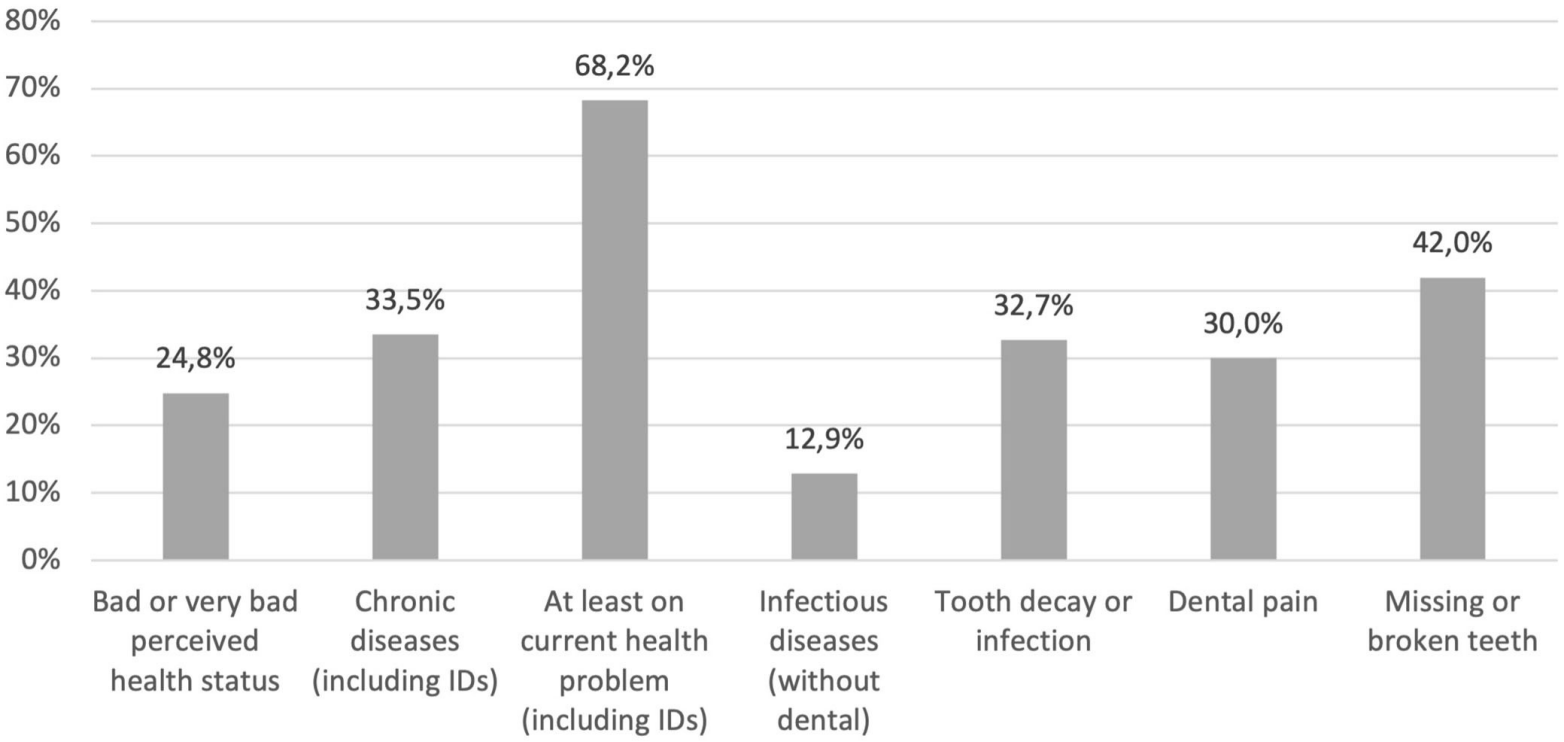
Frequency of health problems and infectious diseases among undocumented migrants in Paris and Bordeaux, Premier Pas survey (*n* = 1.188).

Among the participants (*n* = 1.219), 24.8% declared a poor or very poor perceived health status, 22.2% an average status, and 53.0% a good or very good health status. A chronic health condition or disease (infectious or non-infectious) was reported by 33.5% of respondents (*n* = 1.219). In addition, 68.2% (432/1,189) reported an acute health issue or disease at the time of the survey (Figure 1).

Dental infections (cavities, infection, or pain) concerned 43.2% of the participants: 32.7% cavities or infection, and 30.0% dental pain. As cavities may no longer be an active infection process, this percentage may overestimate the frequency of active infections. Moreover, 42.0% had broken or missing teeth. So, a total of 58.5% had a present or past dental disorder.

Besides these dental issues, 12.9% reported suffering from at least one other current infectious disease: HIV infection (3.5%), chronic hepatitis B infection (3.1%), upper respiratory tract infection (1.7%), skin mycosis (1.2%), skin and soft tissue infection (0.8%), chronic hepatitis C infection (0.8%), urinary tract infection (0.7%), lower respiratory tract infection (0.7%), scabies (0.3%), tuberculosis disease (0.2%), vaginal mycosis 0.6%, and herpes 0.1%. In addition, 7.9% of the respondent reported a skin disorder without the infectious or non-infectious nature being specified.

Regarding HIV, HBV, and HCV infections, 56, 71, and 89%, respectively, reported having been diagnosed after their arrival in France. This was also the case for all the few cases of tuberculosis disease.

Human immunodeficiency virus (HIV) infection was more frequent for participants from sub-Saharan Africa (2.3%) and America than among those of North African origin (0.3%) (*p* < 0.001) (Table 2). Conversely, no statistically significant difference was reported for chronic infection by HBV or HCV according to country of origin. The association with the other covariates is detailed in Table 2.

**Table 2:** Reported prevalence of chronic viral infections (HIV, HBV, or HCV infection) among undocumented migrants in Paris and Bordeaux according to sociodemographic characteristics, Premiers Pas survey (*n* = 1.167).

		**HIV infection**			**Chronic HBV infection**			**Chronic HCV infection**		
	**N**	n/N^*^	**w%^**$**^**	**p^#^**	**n/N**	**w%**	**p**	**n/N**	**w%**	**p**
**Global prevalence**		33/1,188	3.53		36/1,188	3.12%		11/1,188	0.79	
**Sex**	1,220	1,185		0.007	1,185		0.190	1,185		0.233
Male	794	14/767	2.19		27/767	3.70%		9/767	1.03	
Female	426	19/418	6.91		9/418	1.60%		2/418	0.18	
**Age (years)**	1,216	1,181		0.093	1,181		0.130	**1,181**		0.752
18–29	333	6/319	1.89		15/319	4.42%		2/319	0.41	
30–39	443	18/432	5.68		11/432	2.04%		4/432	1.63	
40–82	440	9/430	3.15		10/430	2.85%		5/430	0.31	
**Region of origin**	1,209	1,175		<0.001	1,175		0.02	1,175		0.051
North Africa	363	1,356	0.31		3/356	1.47%		0/356	0.00	
Sub-Saharan Africa	630	13/615	2.27		25/615	3.97%		7/615	1.15	
Other (America, Asia, Europe)	216	19/204	16.51		7/204	1.72%		4/204	0.48	
**Region of origin**	1,209	1,175		<0.001	1,175		0.038	1,175		0.007
North Africa	363	1/356	0.31		3/356	1.47		0/356	0	
Sub-Saharan Africa	630	13/615	2.27		25/615	3.97		7/615	1.15	
Amérique latine et US	80	18/75	33.13		1/75	1.37		0/75	0	
Europe UE	47	1/47	1.22		1/47	0.8		2/47	1.6	
Europe non UE	39	0/35	0.00		2/35	4.99		0/35	0	
Asia	50	0/47	0.00		3/47	1.22		2/47	1.06	
**State medical aid (AME) (actual)**	1,218	1,184		0.006	1,184		0.899	1,184		0.636
Yes	526	22/514	5.54		16/514	3.55		4/514	0.85	
No	692	11/670	1.99		20/670	2.83		7/670	0.75	
**State medical aid (AME) (by the past)**	452	443		0.445	443		0.464	443		0.836
Yes	110	1/106	0.31		2/106	0.27		1/106	3.71	
No	342	7/337	2.62		11/337	2.75		4/337	0.46	
**Perceived health status**	1,219	1,184		0.962	1,184		0.036	1,184		0.271
Very good	239	8/228	4.96		2/228	0.98		1/228	0.63	
Good	368	10/359	3.23		10/359	3.63		2/359	0.087	
Average	293	7/284	2.41		9/284	3.79		2/284	1.38	
Bad	243	6/237	3.59		9/237	3.89		4/237	1.48	
Very bad	76	2/76	5.05		6/76	1.91		2/76	0.73	
**Length of stay in France**	1,215	1,182		0.275	1,182		0.401	1,182		0.343
<3 months	132	3/128	1.69		7/128	1.55		3/128	1.74	
3 months−1 Year	324	8/318	4.58		8/318	4.00		2/318	0.21	
1 year−3 years	314	14/307	4.75		11/307	3.92		3/307	0.59	
3 years−5 years	147	3/144	2.79		4/144	5.15		0/144	0	
More than 5 years	298	5/285	2.67		6/285	1.28		3/285	1.42	
**Type of housing**	1,214	1,181		0.009	1,181		0.469	1,181		0.470
Ordinary	536	21/522	5.74		13/522	2.77		4/522	1.15	
Collective	219	8/215	4.15		6/215	2.22		1/215	0.15	
Makeshift	459	4/444	1.25		17/444	3.91		6/444	0.76	
**Work activity**	1,212	1,178		0.769	1,178		0.073	1,178		0.078
Yes	286	8/278	3.68		4/278	1.34		0/278	0	
No	926	23/900	3.24		32/900	3.63		10/900	0.98	
**Food insecurity**	1,214	1,181		0.145	1,181		0.055	1,181		0.019
Often	321	5/312	1.39		11/312	3.38		7/312	2.29	
Sometimes	448	17/431	5.04		17/431	5.22		2/431	0.084	
Never	445	11/438	4.03		6/438	0.48		2/438	0.15	
**Capacity to carry 5 kg**	1,208	1,175		0.109	1,175		0.264	1,175		0.001
Yes, without difficulty	794	16/766	2.66		18/766	3.08		2/766	0.063	
Yes, with some difficulties	192	10/191	7.75		6/191	2.47		6/191	3.06	
Yes with many difficulties	84	3/84	4.16		5/84	3.37		2/84	4.84	
No	138	3/134	1.95		5/134	3.39		1/134	0.14	
**Place of recruitment**	1,223	1,188		<0.001	1,188		0.475	1,188		0.404
Espace Solidarité d'Insertion	265	0/253	0		6/253	2.1		4/253	1.17	
Associations	406	31/395	5.21		13/395	3.48		2/395	0.58	
Free outdoor clinics (PASS)	194	1/189	0.49		9/189	6		4/189	3.06	
Social security centers (CPAM)	43	0/43	0		3/43	5.24		0/43	0	
Baths/showers	54	0/50	0		0/50	0		1/50	4.13	
Doctor to the worlds centers	61	0/61	0		1/61	0.45		0/61	0	
Point of access to rights	8	0/8	0		0/8	0		0/8	0	
Others	182	1/179	0.15		4,179	2.58		0/179	0	
Planning and child protection center	10	0/10	0		0/10	0		0/10	0	
**Family structure**	1,191	1,159		0.012	1,159		0.701	1,159		0.314
Alone	475	19/458	5.09		12/458	3.72		2/458	0.64	
Alone with child	226	6/221	4.28		9/221	2.56		4/221	1.84	
In couple without children	132	6/129	5.03		3/129	3.89		2/129	1.2	
In couple with children	358	2/351	0.51		12/351	2.57		3/351	0.2	
**Knowledge of French (oral)**	1,222	1,187		0.007	1,187		0.484	1,187		0.477
Very good/quite good	643	15/637	2.67		21/637	3.76		4/637	0.76	
Not very well/poorly or badly	277	3/269	2.14		5/269	0.61		3/269	0.34	
Non-French speaking	302	15/281	7.46		9/281	4.01		4/281	1.34	
**Knowledge of French (reading)**	1,221	1,186		0.002	1,186		0.658	1,186		0.551
Very good/quite good	605	16/597	3.37		20/597	3.73		4/597	0.85	
Not very well/poorly or badly	314	2/308	0.96		7/308	1.35		3/308	0.27	
Non-French reading	302	15/281	7.46		9/281	4.01		4/281	1.34	
**Administrative status**	1,184	1,152		0.794	1,152		0.006	1,152		0.004
Receipt	117	3/113	2.56		7/113	5.21		4/113	2.97	
Residence permit in the past	180	3/177	2.9		5/177	1.23		2/177	2.46	
No residence permit but applied for asylum in the past	75	1/69	0.8		5/69	4.42		0/69	0	
No residence permit and no asylum application in the past	812	22/793	3.51		15/793	2.9		3,793	0.24	
**Reason of migration**	1,036	1,016		<0.001	1,016		0.001	1,016		0.002
Political/security	320	4/309	1.58		20/309	7.74		2/309	0.95	
Family	79	2/78	7.58		1/78	0.71		0/78	0	
Studies	30	1/30	11.43		0/30	0		0/30	0	
Economic	486	9/479	2.33		6/479	1.41		2/479	0.12	
Health	121	13/120	16.47		4/120	3.31		5/120	6.14	
**Income (quintiles)**	1,216	1,183		0.567	1,183		0.083	1,183		0.215
1st Quintile	527	14/512	2.84		21/512	2.84		8/512	0.98	
2d Quintile	172	3/162	2.02		0/162	2.02		2/162	2.24	
3rd Quintile	172	4/170	5.00		7/170	5		0/170	0	
4th Quintile	172	4/167	3.55		4/167	3.55		1/167	0.13	
5th Quintile	173	8/172	6.03		4/172	6.03		0/172	0	
**Pregnancy in France**	417	409		0.17	409		0.641	409		0.523
Yes	71	1/69	4.27%		1/69	2.08%		0/69	0.00%	
No	346	18/340	7.46%		8/340	1.54%		2/340	0.21%	

* *Data filled in without missing data*,

$ *weight percentage*,

# *p-value of chi-square test*.

**Figure 2:**
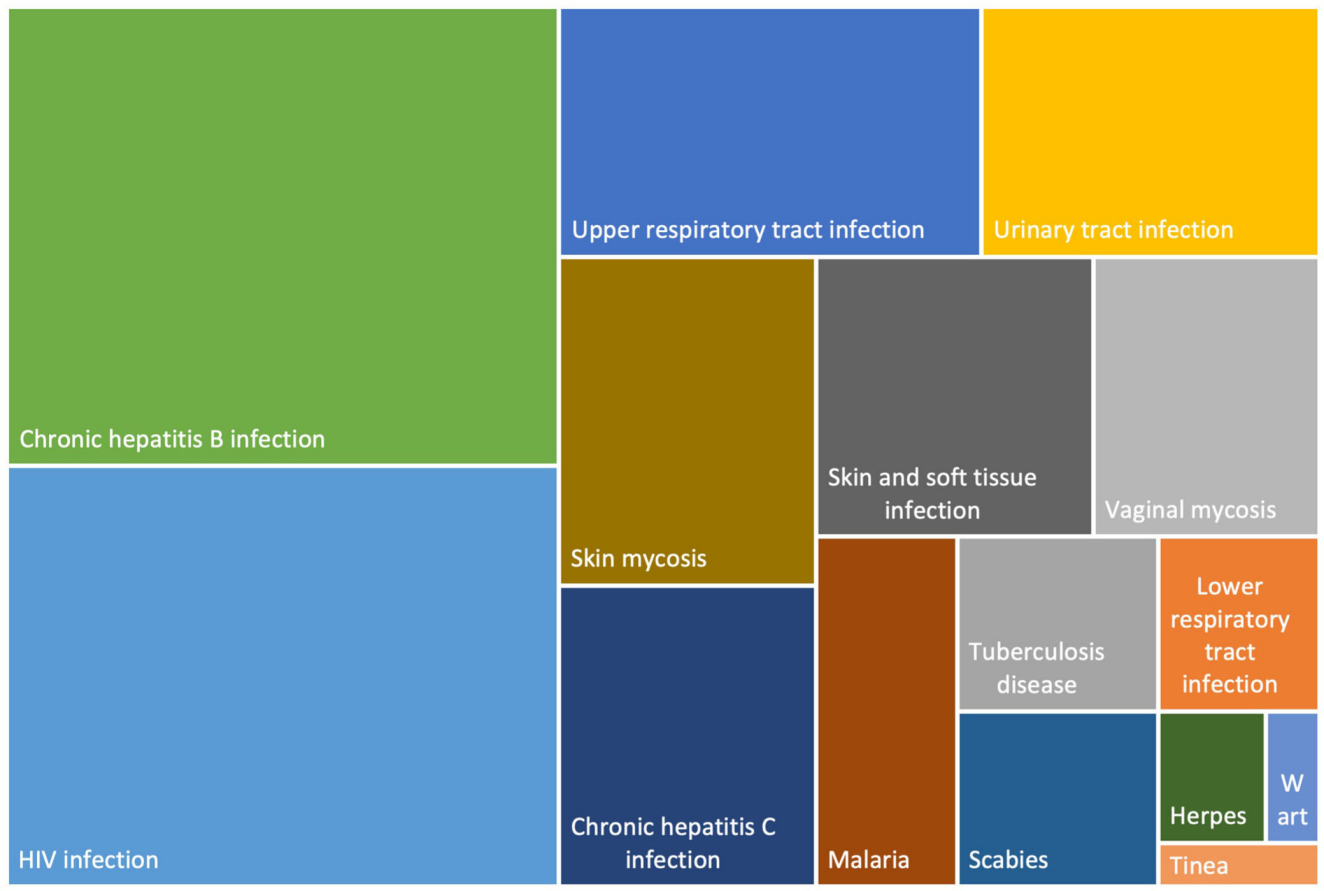
Distribution of infectious diseases among undocumented migrants in Paris and Bordeaux, Premier Pas survey (*n* = 1.188).

Chronic viral infections were more often reported by undocumented migrants from sub-Saharan Africa and Latin America (Table 3). In multivariate analysis, a higher risk of reporting chronic viral infection was observed among people coming from Latin America and Europe, among the food insecure, among those recruited from an association, a free health center, or a social security center, and for single persons with children or couples without children. The association with South American origin is mainly driven by a higher prevalence of HIV among people from South America which will be discussed below (Figure 2). Conversely, people who had migrated for economic reasons and those who had never applied for a residence permit or asylum in France were less likely to report a chronic viral infection (Table 3).

**Table 3:** Reported prevalence of chronic viral infections (HIV, HBV, or HCV infection) among undocumented migrants in Paris and Bordeaux, univariate and multivariate analyses with backward stepwise logistic regression (*p* = 0.05 threshold), Premiers Pas survey (*n* = 1,188).

				**Univariate**			**Multivariate**		
								***N* = 927**	
	n/N^*^	**w%**	**p**	**cOR**	**95%CI**	**p**	**aOR**	**95%CI**	**p**
**Chronic viral infection**	74/1,188	7.07							
**Gender**	1,185		0.41						
Men	29/418	8.4		1.2	[0.74–1.94]	0.467			
Women	45/767	6.6		ref	ref	ref			
**Age (years)**	1,136		0.431						
18–29	21/319	6.1		ref	ref	ref			
30–39	28/387	9.1		1.11	[0.62–1.99]	0.734			
40–82	22/430	6.0		0.77	[0.41–1.42]	0.395			
**Region of origin**	1,167		<0.001						
North Africa	3/353	1.71		ref	ref	ref	ref	ref	ref
Sub-Saharan Africa	42/615	7.0		8.55	[2.63–27.80]	<0.001	8.03	[1.82–35.39]	0.006
Latin America and US	18/75	33.1		36.84	[10.51–129.09]	<0.001	46.11	[9.06–234.50]	<0.001
Europe EU	3/46	2.9		8.14	[1.59–41.60]	0.012	20.71	[2.59–165.66]	0.004
Europe non EU	2/31	7.8		8.05	[1.29–50.10]	0.025	5.12	[0.66–39.83]	0.118
Asia	4/47	1.8		10.85	[2.35–50.12]	0.002			
**State medical aid (AME) (actual)**	1,184		0.084						
Yes	39/514	9.2		1.49	[0.93–2.39]	0.098			
No	35/670	5.5		ref	ref	ref			
**State medical aid (AME) (by the past)**	443		0.758						
Yes	4/106	4.3		0.62	[0.21–1.86]	0.395			
No	20/337	5.7		ref	ref	ref			
**Perceived health status**	1,184		0.878						
Very good	9/228	5.3		ref	ref	ref			
Good	21/359	6.9		1.51	[0.68–3.36]	0.311			
Average	17/284	7.2		1.55	[0.68–3.54]	0.300			
Bad	18/237	8.9		2.00	[0.88–8,455]	0.098			
Very bad	9/76	7.2		3.27	[1.25–8.57]	0.016			
**Length of stay in France**	1,182		0.619						
<3 months	11/128	4.7		1.97	[0.86–4.52]	0.111			
3 months–1 year	18/318	8.8		1.26	[0.60–2.61]	0.543			
1 year–3 years	25/307	8.4		1.85	[0.93–3.70]	0.080			
3 years–5 years	7/144	7.9		1.07	[0.42–2.74]	0.889			
More than 5 years	13/285	5.0		ref	ref	ref			
**Type of housing**	1,181		0.419						
Ordinary	35/522	8.8		ref	ref	ref			
Collective	14/215	6.4		0.97	[0.51–1.84]	0.924			
Makeshift	25/444	5.8		0.83	[0.49–1.41]	0.491			
**Work activity**	1,178		0.311						
Yes	12/278	5.0		0.64	[0.34–1.21]	0.173			
No	59/900	7.4		ref	ref	ref			
**Food insecurity**	1,181		0.087						
Often	19/312	6.2		1.43	[0.74–2.75]	0.283	3.47	[1.31–9.19]	0.012
Sometimes	34/431	10.1		1.89	[1.06–3.37]	0.031	4.45	[1.84–10.82]	0.001
Never	19/438	4.7		ref	ref	ref	ref	ref	ref
**Capacity to carry 5 kg**	1,175		0.126						
Yes, without difficulty	34/766	5.7		ref	ref	ref			
Yes, with some difficulties	19/191	11.8		2.38	[1.32–4.27]	0.004			
Yes with many difficulties	9/84	10.7		2.58	[1.19–5.59]	0.016			
No	9/134	5.5		1.55	[0.73–3.31]	0.258			
**Place of recruitment**	1,188		0.158						
Espace Solidarité d'Insertion	8/253	2.7		ref	ref	ref	ref	ref	ref
Associations	43/395	8.8		3.74	[1.73–8.10]	0.001	3.8	[1.24–11.60]	0.019
Free outdoor clinics (PASS) and Doctor to the worlds centers	14/250	6.9		1.82	[0.75–4.41]	0.187	1.64	[0.47–5.78]	0.438
Social security centers (CPAM)	3/43	5.2		2.3	[0.58–9.02]	0.234	8.9	[1.64–48.24]	0.011
Baths/showers	1/50	4.1		0.63	[0.08–5.11]	0.661	0.88	[0.09–8.49]	0.915
Point of access to rights, planning and child protection center, and others	5/197	2.5		0.8	[0.26–2.48]	0.696	1.34	[0.32–5.63]	0.694
**Family structure**	1,159		0.096						
Alone	32/458	9.2		1.68	[0.90–3.16]	0.105			
Alone with child	18/221	8.6		1.99	[0.98–4.03]	0.057			
In couple without children	9/129	8.0		1.68	[0.72–3.94]	0.233			
In couple with children	15/351	3.1		ref	ref	ref			
**Knowledge of French (oral)**	1,187		0.010						
Very good/quite good	38/637	6.8		ref	ref	ref			
Not very well/poorly or badly	10/269	3.0		0.61	[0.30–1.24]	0.171			
Non-French speaking	25/281	12.3		1.54	[0.91–2.60]	0.108			
**Knowledge of French (reading)**	1,186								
Very good/quite good	38/597	7.5		ref	ref	ref			
Not very well/poorly or badly	11/308	2.5		0.54	[0.27–1.08]	0.083			
Non-French reading	25/281	12.3		1.44	[0.85–2.43]	0.177			
**Administrative status**	1,152		0.627						
Receipt	13/114	10.5		ref	ref	ref			
Residence permit in the past	9/177	5.9		0.42	[0.17–1.01]	0.052			
No residence permit but applied for asylum in the past	6/69	5.2		0.74	[0.27–2.05]	0.562			
No residence permit and no asylum application in the past	37/792	6.3		0.38	[0.20–0.74]	0.004			
**Reason of migration**	1,016		0.002						
Political/security	26/309	10.3		0.56	[0.29–1.07]	0.078	0.51	[0.20–1.20]	0.159
Family	3/78	8.3		0.24	[0.07–0.86]	0.028	0.3	[0.07–1.30]	0.108
Studies	1/30	11.4		0.21	[0.03–1.64]	0.136	0.29	[0.03–2.58]	0.266
Economic	17/479	3.9		0.22	[0.11–0.45]	<0.001	0.19	[0.07–0.49]	0.001
Health	17/120	21.0		ref	ref	ref	ref	ref	ref
**Income (quintiles)**	1,183		0.323						
1st Quintile	38/512	6.2		1.07	[0.55–2.10]	0.846			
2d Quintile	5/162	4.3		0.42	[0.15–1.23]	0.115			
3rd Quintile	11/170	12.2		0.92	[0.40–2.15]	0.852			
4th Quintile	8/167	6.5		0.67	[0.27–1.69]	0.396			
5th Quintile	12/172	8.6		ref	ref	ref			

*HIV, HIV infection; HBV, hepatitis B virus; HCV, hepatitis C virus; w%, weighted percentage; cOR, crude odds ratio; aOR, adjusted odds ratio; EU, European Union; ref., Reference category*.

* *Number of completed data after removal of missing data for the variable*.

In terms of prevention of infectious diseases, 54.7% reported having been tested for HIV since arriving in France and 46.2% have had a chest X-ray for tuberculosis disease screening. Catch-up vaccination was started in only 35.4% of participants.

## Discussion

This original study on the frequency of infectious diseases among undocumented migrants in Bordeaux and Paris highlights the healthcare needs of this vulnerable population.

The study reports a high prevalence of dental abnormalities and infections, a public health issue that is often neglected in studies of migrant health. A review of studies analyzing the oral health status of migrants from middle-income and low-income countries to Europe reported a high prevalence of caries experience and lack of dental health in the host countries (22). A recent study has been conducted in France in a health center offering dental consults to migrants (23). In this study, of the migrants who were assessed by a dentist, 72.3% of migrants had cavities and 59% needed extensive oral care.

The concern around infectious diseases and migration is an issue, as migrants are disproportionately exposed to infectious and tropical diseases (4). This includes an increased risk of COVID-19, HIV, hepatitis B, tuberculosis, schistosomiasis, and malaria (11, 24, 25). This issue is linked to the epidemiology of infectious diseases in the countries of origin, but also to poor conditions of migration resulting in exposure to infectious diseases and, in some countries, to the lack of access to a healthcare system and to insurance coverage among migrants (12, 17, 26, 27). Existing evidence from different countries highlights the difficulties that migrants face in accessing health services, which contributes to the burden of excess morbidity and mortality faced by migrants (9, 28–30).

Participants frequently reported common infectious diseases including skin and soft tissue infections, upper air tract infections, urinary, and gynecological infections (31–34). This is in line with the findings of other work carried out among the migrant populations. These infections may be linked to poor living conditions and sexual vulnerability that some migrants in deprived situations may experience.

Migrants are a key population affected by HIV (8). While the prevalence of HIV infection is linked to the prevalence in the country of origin, it has been shown that precariousness and poor migration conditions increase the risk of acquiring HIV after arrival in the country of destination (35). It is likely that the prevalence of HIV infections has been overrepresented here in connection with better social and healthcare support for this population, and therefore a higher chance of being met in the partner facilities. France is a French-speaking country. Migration from South America, the majority of which is Spanish speaking, is limited in number and most often concerns migration for professional or family reasons of people in a regular administrative situation. On the other hand, France is home to a community of transgender sex workers women often living with HIV (36). Some of them are accompanied by a part of the Parisian investigation structures. Despite these selection biases, the study's sample remains original in terms of its size and the diversity of the recruitment sites used. It is also important to learn that most HIV infections were diagnosed after their arrival on the territory. This should be weighed against the risk of being infected by HIV after arrival, which is estimated in France at up 50% (35).

Tuberculosis is a rare disease in Europe where migrants represent a large part of residual cases (6). Tuberculosis in migrants is commonly the result of reactivation of latent infection with the bacteria *Mycobacterium tuberculosis* acquired outside the host country. Without treatment, it is possibly a serious illness. It is also the most advanced disease that can become contagious. An early diagnosis and access to healthcare are therefore essential to prevent complications and secondary transmissions. Tuberculosis can be detected before the onset of the disease by screening for latent tuberculosis infection. Our results confirm the relative rarity of visible diagnosed tuberculosis, without prejudging the frequency of latent forms.

Parasitic diseases are frequent among migrants from endemic countries and can reveal themselves through symptoms, in particular digestive symptoms. Malaria is an important febrile disease that is mainly observed in travelers visiting migrants to non-endemic countries (37). Other parasitic diseases can be asymptomatic but carry significant morbidity, such as schistosomiasis and strongyloidiasis (38). It is therefore likely that these infections are underestimated in this study, which is declarative because they are asymptomatic and not diagnosed.

Similarly, HTLV1 infection, which is most often asymptomatic, is largely unrecognized and underscreened among migrants in host countries (39). It was therefore predictable that it would not be measured in this study. However, it is an infection that can be transmitted through sexual intercourse or breastfeeding and can be complicated by leukemia or severe neurological damage.

Even though our study took place just before the pandemic, people living in precarious or overcrowded conditions, including undocumented migrants, have been more severely impacted by the COVID-19 pandemic (40, 41). As with other vaccine-preventable diseases, ensuring equitable and unconditional access to vaccination uptake and ensuring hygiene is the first step in preventing these respiratory viruses.

The association between food insecurity and increased incidence of chronic viral infections is of concern. In the same study, it was shown that food insecurity was a key factor in the likelihood of developing post-traumatic stress disorder (42). Systematic detection of hunger and insecurity in terms of access to sufficient food in quantity and quality should be systematic.

This survey has several limitations. Despite the resources deployed, the language barrier may have remained an obstacle in a small proportion of cases. However, the main countries of origin were well-represented with an adapted interpreting offer. Despite the inclusion of key places and structures in the support of migrants in Paris and Bordeaux, the sample may misrepresent the migrants furthest from the structures or not be visible in the public space. Furthermore, as health and social support contribute to access to care and screening, the prevalence of infectious diseases may have been overestimated. The declarative health status is an important limitation of this work as it exposes classification bias and lack of awareness of undiagnosed pathologies. Moreover, a study based on declarative status is not as valuable as a study based on a thorough medical examination, especially in the absence of biological and radiological screening for asymptomatic conditions.

This survey also has many strengths. The interviews were conducted in the migrant's language by interviewers trained in public health and listening. Acceptability was good. The size of the sample is large. The diversity of the structures surveyed contributes to the representativeness of the sample. Systematic questioning on all major symptoms and disease families reduces the risk of reporting bias.

## Conclusion

This survey conducted in France on the health of undocumented migrants highlights the frequency of infectious diseases among undocumented migrants. Systematic screening for symptoms suggestive of infectious diseases offers a comprehensive health check-up within 4 months of arrival, vaccine catch-up, and the extensive use of diversified prevention tools in an unconstrained framework are key elements of risk reduction (43). This check-up contributes to the diagnosis of combined infections. The precise contours of the deployment of this health check-up in the various places and structures welcoming newcomer migrants are still under construction. The identification of factors associated with the prevalence of chronic viral infections is thus likely to contribute to the prioritization of people and structures where the health check-up should be deployed. Given the links with food insecurity, it seems interesting to deploy this offer within solidarity grocery shops and social structures. In addition, the already existing link between the social security centers and the health examination centers should be strengthened.

Early diagnosis and case management of infectious diseases are compulsory. Access to healthcare for undocumented migrants is thus essential. Information and the implication of community actors about infectious diseases lead to the empowerment of migrants in terms of health, in addition to protecting them. Prevention programs should ensure that all migrants are provided with free and facilitated access to repeated HIV testing, linkage to care and treatment if they are HIV positive, and pre-exposure prophylaxis for HIV-negative migrants if relevant. The implementation of a health rendezvous offered to all and integrating a broad clinicobiological screening of prevalent infectious diseases is advisable (44). To reach migrants who are far from the healthcare system, mobile public health teams would be recommended. More generally, the prevention of infectious diseases and their consequences among migrants is based on a reduction of poverty and social exclusion, equitable access to healthcare, prevention measures, and social services, and a culturally sensitive patient-centered approach (45). In this respect, the recent setbacks in French law on access to healthcare for undocumented migrants are worrying (46). A complementary infectious diseases seroprevalence survey of a representative sample of undocumented migrants would be useful to highlight these findings and clarify the objectives of the health check-ups. Infectious disease screening and prevention programs are likely to have an important role in improving the health and social determinants of health for newly arrived and settled undocumented migrants.

## Data availability statement

The raw data supporting the conclusions of this article will be made available by the authors, without undue reservation.

## Ethics statement

The studies involving human participants were reviewed and approved by Institute for Research and Documentation in Health Economics. Written informed consent for participation was not required for this study in accordance with the national legislation and the institutional requirements.

## Author contributions

JW, FJ, and PD: conceptualization. NV, SM, and PD: methodology and data processing. NV: writing—original draft. All authors writing—review and editing.

## Funding

This work was funded by French National Research Agency (Agence nationale de la recherche, ANR).

## Conflict of interest

The authors declare that the research was conducted in the absence of any commercial or financial relationships that could be construed as a potential conflict of interest.

## Publisher's note

All claims expressed in this article are solely those of the authors and do not necessarily represent those of their affiliated organizations, or those of the publisher, the editors and the reviewers. Any product that may be evaluated in this article, or claim that may be made by its manufacturer, is not guaranteed or endorsed by the publisher.
